# Expanding 5q-SMA Newborn Screening in Latin America: A Brazilian Model for National and Regional Implementation

**DOI:** 10.3390/ijns12030050

**Published:** 2026-07-07

**Authors:** Diogo Nani, Rodrigo Holanda Mendonça, Felipe Franco da Graça, Vitoria Regia Pereira Pinheiro, Mirella Carneireiro, Bruna Glaucia Farah, Marcondes Cavalcante França, Carmen Silvia Gabetta, Graziela Polido, Cristina Iwabe, Frederico Monfardini, Alulin Tácio Quadros Santos Monteiro Fonseca, Paulo Breinis, Carmela Maggiuzzo Grindler, Carlos Eugenio Fernandez de Andrade, Athene Maria de Marco França Mauro, Edmar Zanoteli, Wilson Marques, Léa Maria Zanini Maciel, Acary Souza Bulle Oliveira, Maria da Penha Ananias Morita, Edward Yang, Vanessa Luiza Romanelli Tavares

**Affiliations:** 1 Newborn Screening Reference Service, Instituto Jô Clemente (IJC), São Paulo 04040-033, Brazil; diogo.nani@ijc.org.br (D.N.); mirella.carneireiro@ijc.org.br (M.C.); bruna.farah@ijc.org.br (B.G.F.); edward.yang@ijc.org.br (E.Y.); 2 Department of Neurology, Faculty of Medicine, University of São Paulo (FMUSP), São Paulo 05403-010, Brazil; rodrigo.holanda@hc.fm.usp.br (R.H.M.); graziela.polido@hc.fm.usp.br (G.P.); edmar.zanoteli@usp.br (E.Z.); 3 Department of Neurology, Faculty of Medical Sciences, State University of Campinas (UNICAMP), Campinas 13083-888, Brazil; fgraca@unicamp.br (F.F.d.G.); mcfjr@unicamp.br (M.C.F.J.); crisiwabe@hotmail.com (C.I.); 4 Integrated Center for Oncohematological Research in Childhood, State University of Campinas (CIPOI/UNICAMP), Campinas 13083-894, Brazil; vitoria@unicamp.br (V.R.P.P.); sgabetta@unicamp.br (C.S.G.); 5 Hospital Israelita Albert Einstein, São Paulo 05652-900, Brazil; frederico.monfardini@einstein.br; 6 Paulista School of Medicine, Federal University of São Paulo (UNIFESP), São Paulo 04039-060, Brazil; alulin.fonseca@unifesp.br (A.T.Q.S.M.F.); acary.bulle@unifesp.br (A.S.B.O.); 7 Department of Neurology, School of Medicine of ABC (FMABC), Santo André 09060-650, Brazil; paulobreinis@uol.com.br; 8 Irmandade da Santa Casa de Misericórdia de São Paulo, São Paulo 01221-010, Brazil; 9 Secretaria de Estado da Saúde de São Paulo, São Paulo 01027-000, Brazil; cgrindler@saude.sp.gov.br; 10 Secretaria Municipal da Saúde de São Paulo, São Paulo 01509-020, Brazil; carlosfernandez@prefeitura.sp.gov.br (C.E.F.d.A.); ammauro@prefeitura.sp.gov.br (A.M.d.M.F.M.); 11 Ribeirão Preto Medical School, University of São Paulo (FMRP-USP), Ribeirão Preto 14049-900, Brazil; wmjunior@fmrp.usp.br (W.M.J.); leamaciel@hcrp.usp.br (L.M.Z.M.); 12 Hospital de Base, Faculty of Medicine of São José do Rio Preto (FAMERP), São José do Rio Preto 15090-000, Brazil; mpenha1960@hotmail.com

**Keywords:** spinal muscular atrophy, newborn screening, 5-SMA, *SMN1*, *SMN2*, real-word evidence, Brazil, Latin America

## Abstract

5q spinal muscular atrophy (5q-SMA) is a leading genetic cause of infant mortality. Presymptomatic intervention with disease-modifying therapies significantly improves motor outcomes, but effectiveness depends on early detection through newborn screening (NBS). Despite global 5q-SMA NBS expansion and recent Brazilian federal legislation, regional disparities and a lack of systematic monitoring hinder access to timely diagnosis and care. This study addresses these gaps by evaluating a statewide pilot program in São Paulo. We used multiplex real-time PCR to detect *SMN1* exon 7 deletions in dried blood spots, confirming *SMN1/2* copy numbers via MLPA in positive cases. Under real-world conditions, timeliness key performance indicators were evaluated to assess operational efficiency. 194,714 newborns were screened with 14 positive cases, yielding a prevalence of 1:13,908. First-tier results and treatment initiation occurred at a median of 10.8 and 28 days of life, respectively. Notably, 78.6% of patients had two *SMN2* copies, of which approximately half were symptomatic by the first evaluation, highlighting the critical need for rapid screening to prevent irreversible motor decline. Screening achieved 100% specificity. This pilot demonstrates the feasibility of 5q-SMA NBS within the Brazilian public health system, providing essential evidence to overcome logistical and socioeconomic barriers and support nationwide expansion.

## 1. Introduction

5q spinal muscular atrophy (5q-SMA) is characterized by the progressive loss of motor neurons in the anterior horn of the spinal cord (OMIM #253300, #253550, #253400, #271150). As one of the leading genetic causes of infant mortality worldwide [1,2,3], it has a birth prevalence of approximately 1 in 10,000 live births [4,5]. Disease progression reflects irreversible motor neuron loss, manifesting as symptom onset or clinical worsening followed by progressive muscular atrophy [6]. It is caused by insufficient levels of the survival motor neuron (SMN) protein, essential for motor neuron maintenance and primarily encoded by the *SMN1* gene, whose homozygous absence leads to markedly reduced SMN production [7]. The paralogous gene *SMN2* modulates disease severity by producing limited amounts of functional SMN protein, as alternative splicing frequently excludes exon 7 from *SMN2* transcripts [8].

Clinical trials (SPR1NT, RAINBOWFISH, and NURTURE), along with real-world evidence have shown that presymptomatic treatment with disease-modifying therapy (DMT) results in substantial motor improvements and prevents progression to severe phenotypes, especially when initiated early [9,10,11,12,13]. These findings underscore the vital role of newborn screening (NBS) to enable timely treatment.

NBS programs based on real-time polymerase chain reaction (real-time PCR) for the detection of *SMN1* exon 7 homozygous absence, which accounts for approximately 95% of spinal muscular atrophy cases, have been validated and implemented in several countries with high clinical sensitivity and clinical specificity [14,15,16]. By 2023, 31 countries had implemented official NBS programs, with projections indicating 18% of newborns worldwide to be screened by 2028 [17].

Historically, patients have faced considerable delays between symptom onset and molecular confirmation, resulting in substantial motor function loss before treatment initiation [18]. In Brazil, these delays may be even more pronounced due to restricted access to molecular diagnostics and a shortage of rare disease specialists. In 2020, there were only 332 medical geneticists nationwide (approximately one per 665,000 individuals) [19]. A federal law regulating NBS in Brazil aims to expand the panel from six to over 50 target diseases, including 5q-SMA [20]. Nonetheless, implementation effectiveness varies widely across regions and states with overrepresentation of research and service capacity in the Southeast, while the North and the Northeast regions remain under-resourced [21,22].

According to the Ministry of Health, many public health policies do not have program performance indicators systematically monitored and fail to close the feedback loop between evidence generation and decision-making, and rare disease policies tend to emerge reactively instead of following a systematic monitoring of screening outcomes and follow-up processes [23,24]. For 5q-SMA NBS, this means that evaluation must be planned, resourced, and integrated into governance mechanisms from the outset. The Ministry also stresses that evaluations should be usable and that results must be translated into decision-making, especially in decentralized systems [24].

Therefore, we conducted a prospective, non-interventional clinical pilot study aimed at structuring, operationalizing, and evaluating the workflow of a statewide NBS program for 5q-SMA in São Paulo State, Brazil. The study evaluated key performance indicators across the NBS process under real-world conditions, including time from specimen collection to confirmatory testing and treatment initiation, and the short-term follow-up performance to the Specialized Reference Centers for Spinal Muscular Atrophy (Centros Especializados de Referência de Atrofia Muscular Espinhal—CERAME). The study cohort consisted of dried blood spots (DBS) specimens screened in a timely manner at the Instituto Jô Clemente (IJC), one of the three Reference Services for Neonatal Screening (RSNS) in the state of São Paulo, and responsible for approximately 68% of NBS in the state [25]. The two other RSNS are located in Campinas and Ribeirão Preto, where samples also came from. Data generated through this process also allowed a real-world estimation of birth prevalence and screening outcomes of 5q-SMA within the NBS program.

## 2. Materials and Methods

### 2.1. Ethical Considerations

The study was conducted in accordance with the Declaration of Helsinki and approved by the National Commission for Research Ethics (CONEP), under approval number (CAAE) 56631522.0.0000.8647, approved on 7 December 2022. An informational leaflet attached to the sample collection card was provided to parents or legal guardians, describing data confidentiality in compliance with the Brazilian General Data Protection Law (LGPD) and the objectives of the project. Written informed consent was obtained only from newborns with confirmed 5q-SMA whose families participated in subsequent clinical follow-up and data collection.

### 2.2. Cohort and Referral Pathway

Newborns delivered within the coverage of the public RSNS network in São Paulo were included in the 5q-SMA NBS pilot program. This cohort encompasses the capital city and regions representative of the broader state population. The screening algorithm and the multidisciplinary team at IJC are described in detail elsewhere [26].

Upon identification of a screen-positive result, parents or legal guardians were contacted by the Active Search department. This team was responsible for coordinating confirmatory testing, scheduling neuropediatric evaluations at the CERAME, and providing genetic counseling, ensuring a seamless transition from screening to clinical assessment.

### 2.3. Assessment of SMN1 and SMN2 Copy Number

In first-tier testing, real-time PCR was used to detect homozygous absence of *SMN1* exon 7 from DBS samples on filter paper (Guthrie paper); carrier status was not evaluated.

DNA was extracted from 3.2 mm diameter DBS punches as follows: 150 μL of 0.5% DBS wash buffer (1X PBS [Thermo Fisher Scientific, Waltham, MA, USA; J61196.AP] and 0.5% Thesit [Sigma-Aldrich, St. Louis, MO, USA; 88315]) was added to each sample or control well. The plate was sealed and centrifuged at 2400 RPM for 1 min, then shaken at 1500 RPM for 30 min. After shaking, the plate was centrifuged again at 2400 RPM for 1 min, and the supernatant was discarded. Subsequently, 150 μL of nuclease-free water was added to each sample well and promptly discarded. Next, 5 μL of DNA Extract All Reagent Lysis Solution (Thermo Fisher Scientific, Waltham, MA, USA; 4402616) was added to each well. The plate was resealed, centrifuged at 2400 RPM for 1 min, and incubated in a thermocycler at 95 °C for 5 min. After incubation, the plate was centrifuged once more at 2400 RPM for 1 min and allowed to return to room temperature before the seal was removed. Finally, 35 μL of TE buffer (Thermo Fisher Scientific, Waltham, MA, USA; 12090015), supplemented with Tween-20 (Thermo Fisher Scientific, Waltham, MA, USA; 85113) at a 1:1000 dilution, was added to each well. The plate was sealed again and centrifuged at 2400 RPM for 1 min. Extracted DNA was either used immediately for real-time PCR reactions or stored at 2–8 °C for up to 24 h.

The TaqMan™ SCID/SMA Plus Assay (Thermo Fisher Scientific, Waltham, MA, USA; A47928) was employed in a multiplex real-time PCR format, together with amplification of primary immunodeficiency markers KREC and TREC. Procedures were performed according to the manufacturer’s instructions, with in-house validation for a 20 μL reaction volume using the QuantStudio 7 Pro Real-Time PCR System (Thermo Fisher Scientific, Waltham, MA, USA). The real-time PCR conditions for the reaction mixture were as follows: (1) initial denaturation/enzyme activation at 95 °C for 20 s; (2) 40 cycles of denaturation at 95 °C for 1 s and annealing/extension at 60 °C for 20 s. In accordance with the IJC NBS program protocol, samples with screen-positive results for 5q-SMA were retested following new DNA extraction from the same DBS specimen.

Newborns with screen-positive results in first-tier testing were recalled for buccal swab collection, followed by Multiplex Ligation-dependent Probe Amplification (MLPA) analysis performed by an external laboratory to confirm absence of *SMN1* exon 7 and determine *SMN2* exon 7 copy number in the second tier. To ensure the accuracy of MLPA results, digital PCR (dPCR), also performed by an external laboratory, was used for confirmatory purposes using blood samples.

### 2.4. Clinical Evaluation

All newborns with confirmed diagnosis were referred to the CERAME nearest to their residence for clinical follow-up and comprehensive guidance, and were monitored by the NBS service for two consultations or until initiation of DMT. The first specialist evaluation was performed after the screen-positive result, and the second after confirmatory diagnosis. Five CERAME operate within the public health system of São Paulo State: three in the city of São Paulo, one in Campinas, and one in Ribeirão Preto.

### 2.5. Statistical Analysis

Birth prevalence and corresponding 95% confidence intervals were calculated assuming a Poisson distribution for confirmed cases, with exact limits derived from the chi-square distribution.

The distribution of continuous variables was assessed using descriptive and graphical methods, including Q–Q plots and histograms, with skewness and kurtosis calculated to characterize distributional asymmetry and tail behavior. In analyses with small sample sizes, normality was additionally evaluated using the Shapiro–Wilk test.

Time intervals across sample collection networks (Campinas, São Paulo, and Ribeirão Preto) were compared using a nonparametric bootstrap approach. Pairwise differences between centers were estimated as differences in medians on the original time scale (days or hours). Empirical 95% confidence intervals were derived from 10,000 bootstrap resamples using the percentile method, and statistical significance was assessed using two-sided bootstrap-based *p*-values under the null hypothesis of no median difference, with a small-sample correction applied to avoid zero *p*-values.

All analyses were performed in R (version 4.5.1).

## 3. Results

### 3.1. Time Indicators Across the NBS Pathway

Our cohort included all 85 public sample-collection sites in the city of São Paulo within the IJC NBS coverage area, as well as 16 sites in the Campinas and 31 in the Ribeirão Preto NBS coverage areas, totaling 132 sites statewide. Over two years and three months of the NBS pilot program, 194,714 newborns were screened, most from the city of São Paulo (183,046; 94.0%), followed by Campinas (10,274; 5.3%) and Ribeirão Preto (1394; 0.7%) areas. 14 cases (six females and eight males) were confirmed with 5q-SMA, corresponding to a birth prevalence of 1:13,908 live births (95% confidence interval: 1:8289 to 1:25,440). No screen-inconclusive or false-positive screening results were observed. The identification of the *SMN1* exon 7 homozygous absence by real-time PCR demonstrated 100% clinical specificity and 95% clinical sensitivity, considering the presumed distribution of these pathogenic genotypes.

**Figure 1:**
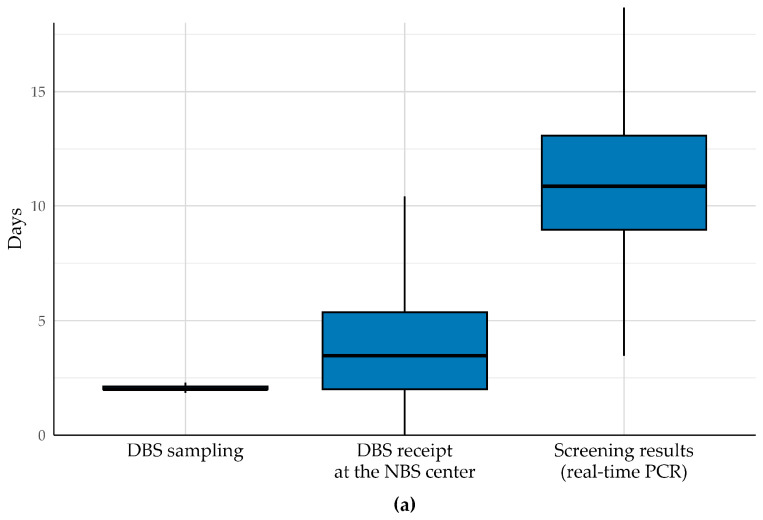

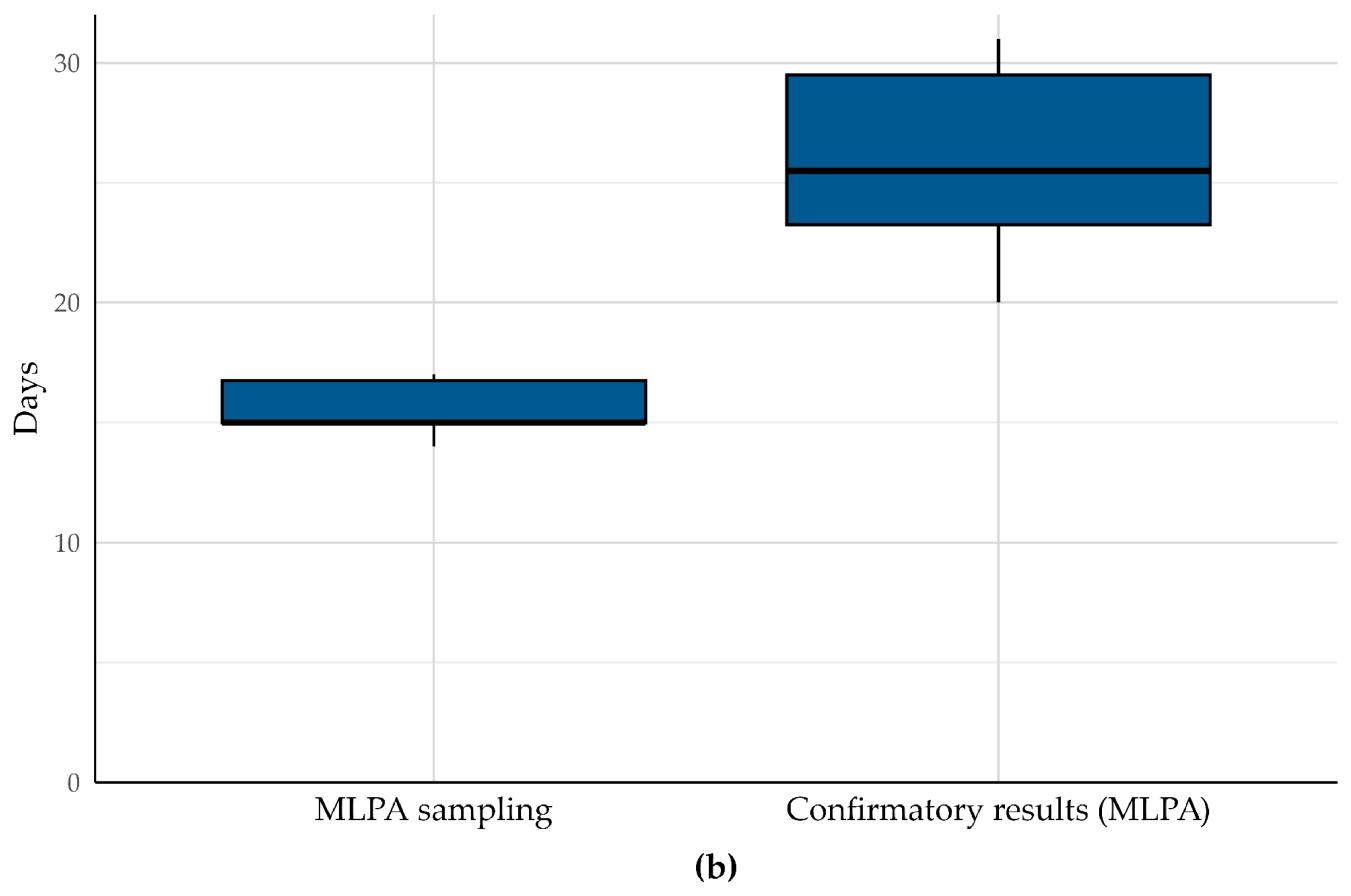
Timeliness key performance indicators. (**a**) First-tier screening by real-time PCR (*n* = 194,714). (**b**) Confirmatory testing by MLPA (*n* = 13); patient 6 was excluded due to diagnosis prior to the screening program. Boxplots show the median and interquartile range (IQR), with whiskers extending to 1.5 × IQR.

**Figure A1:**
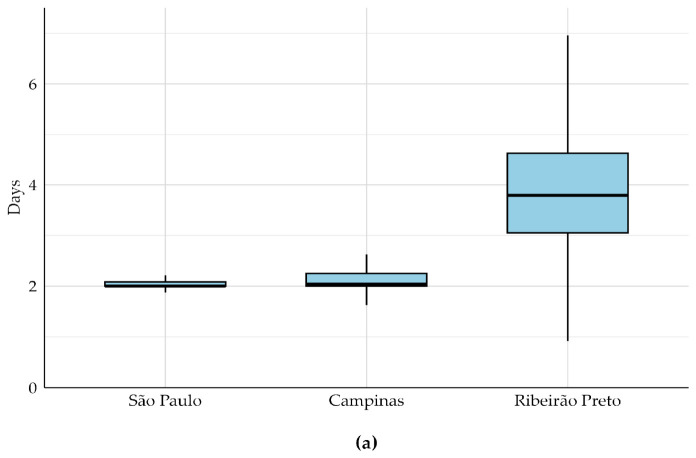

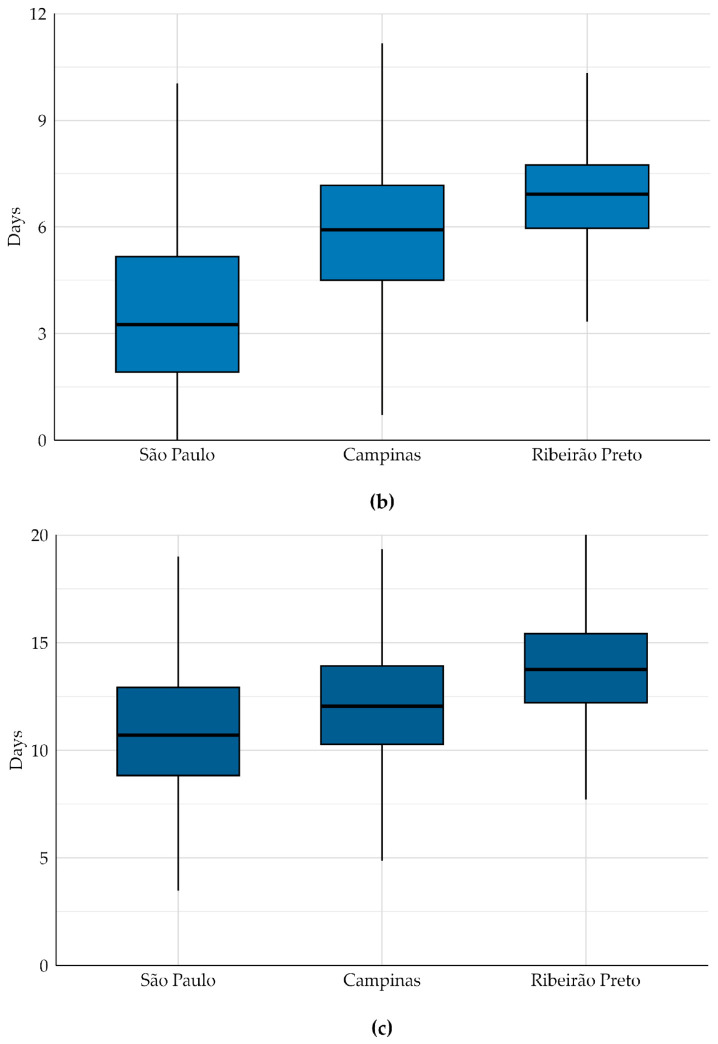
First-tier timeliness indicators across the state of São Paulo, including the city of São Paulo (*n* = 183,046), Campinas (*n* = 10,274), and Ribeirão Preto (*n* = 1394). (**a**) DBS sampling; (**b**) DBS receipt at the NBS center; and (**c**) screening results by real-time PCR. Boxplots depict the median and interquartile range (IQR), with whiskers extending to 1.5 × IQR. Pairwise nonparametric bootstrap comparisons of median times demonstrated statistically significant differences across all centers (bootstrap *p* < 0.001). Differences in DBS sampling time between São Paulo and Campinas were minimal, whereas Ribeirão Preto showed longer sampling times than both centers. Shorter turnaround times were consistently observed in the city of São Paulo for DBS receipt (2.75–3.67 days shorter) and real-time PCR result reporting (1.38–3.06 days shorter) compared with Campinas and Ribeirão Preto.

The timeline up to the screening report showed a median of 2 (IQR: 2–2.13) days of life (DoL) for DBS sampling, and 3.46 days (IQR: 2–5.38) from sampling to receipt at the IJC. Overall, first-tier screening results were reported at a median of 10.83 DoL (IQR: 8.96–13.08) (Figure 1a). Across RSNS coverage areas, a consistent gradient was observed, with shorter times in the city of São Paulo, intermediate times in Campinas, and longer delays in Ribeirão Preto, except for the differences found between São Paulo and Campinas for DBS sampling. Data for each RSNS coverage area can also be seen on Figure A1a–c. For the confirmatory test, samples for MLPA were collected at a median of 15 (IQR: 15–17) DoL, and results were reported at 25.5 (IQR: 23.25–30) DoL. No differences were observed between São Paulo (*n* = 11) and Campinas (*n* = 2), while no positive screening results were identified in Ribeirão Preto (Figure 1b). All MLPA results were confirmed by dPCR.

**Figure 2:**
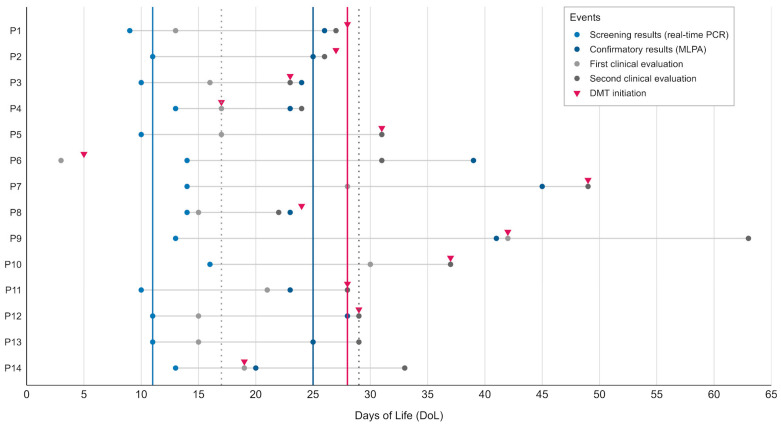
Patient-level timeline of 5q-SMA identification and clinical management. Vertical lines represent the median timing of each step in days of life (DoL): 11 DoL for first-tier result reporting (light blue), 17 DoL for the first clinical evaluation (dashed light gray), 25 DoL for confirmatory result reporting (blue), 28 DoL for disease-modifying therapy (DMT) initiation (magenta), and 29 DoL for the second clinical evaluation (dashed gray). Data from Patient 6 were excluded from all median calculations.

The patient-level timeline of the 14 newborns with screen-positive results is presented in Figure 2. Owing to family history and early symptoms, the diagnosis for patient 6 was pursued and DMT was initiated before identification through the NBS pathway; consequently, data from this patient were excluded from all the median calculation. Median reporting times were 11 DoL (IQR: 10–13) for first-tier screening and 25 DoL (IQR: 23–30) for confirmatory results. The confirmatory test was reported at a mean of 10.8 ± 3.5 days following sample collection. The first clinical evaluation occurred after a screen-positive result was reported, at a median of 17 DoL (IQR: 15–22.8). Patient 2 did not undergo the first clinical evaluation due to immediate neonatal admission to the intensive care unit for complications unrelated to 5q-SMA. The second clinical evaluation occurred at a median of 29 DoL (IQR: 26–33). DMT was initiated at a median of 28 DoL (IQR: 23.8–32.5) (Figure 2).

Unforeseen delays from the MLPA supplier and families’ limited contact access due to socioeconomic constraints contributed to a longer time to DMT initiation for patients 7, 9, and 10. Patient 7 progressed from an asymptomatic status to respiratory difficulties, including paradoxical breathing and tongue fasciculations, as well as hypotonia and reduced movements of the lower limbs, culminating in frog-leg position. In contrast, patient 9 did not exhibit clinical worsening compared to the initial evaluation, which had already shown lower limb hypotonia, mild axial hypotonia, patellar hyporeflexia, slightly reduced extremity movements, and tongue fasciculations since the first clinical evaluation. Patient 10 was asymptomatic from the first clinical evaluation to the time of DMT initiation.

Age at first DMT administration varied widely across patients, with a median of 28 DoL (19–49) (Figure 2). Patients 4, 9, and 14 received DMT at their first evaluation at the CERAME through a compassionate-use donation facilitated by families, due to the severity of their clinical presentation; all were subsequently confirmed to have two *SMN2* copies. The remaining nine patients who were eligible for treatment received DMT after the confirmatory result. Patient 13 was not eligible for DMT under Brazilian jurisdiction.

Risdiplam was the initial therapy in 92.3% of patients (12/13), with at least six individuals subsequently receiving Zolgensma by the time of writing this paper; in contrast, 7.7% of patients (1/13) received Nusinersen as first-line treatment.

### 3.2. SMN2 Copy Number and Clinical Presentation

In our cohort, 78.6% (11/14) of patients harbored two *SMN2* copies, 14.3% (2/14) harbored three copies, and 7.1% (1/14) four copies. Among patients with three or four *SMN2* copies, no symptoms were exhibited during the time of the study. 50% (5/10; 1 missing data) of patients with two *SMN2* copies were symptomatic at first clinical evaluation.

64.3% (9/14) of the cases manifested symptoms within the first two months of life, consistent with 5q-SMA type 1. Additionally, 28.6% (4/14) of patients evolved from asymptomatic to symptomatic status between the first and second clinical evaluations, over a period ranging from one to three weeks while awaiting the MLPA confirmatory test. 28.6% (4/14) of patients were treated while still asymptomatic (Table 1).

### 3.3. CHOP-INTEND Scores and Clinical Presentation

**Figure 3:**
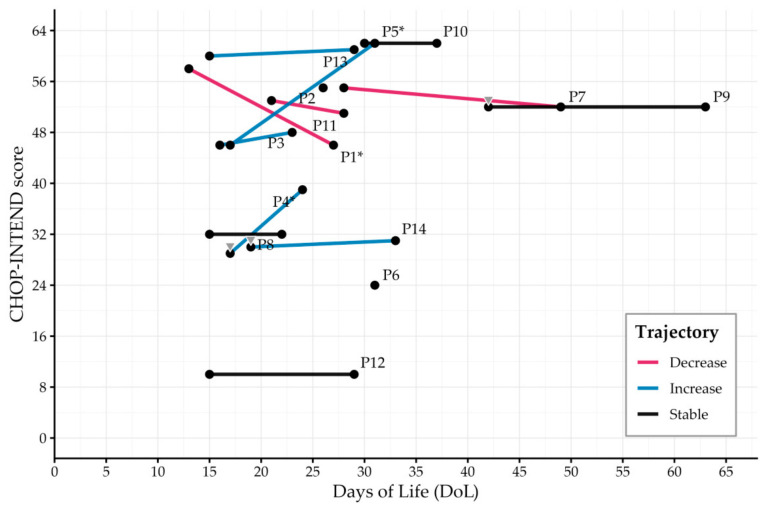
Change in CHOP-INTEND score between the first and second clinical evaluations. Lines represent individual patients and are color-coded according to the direction of change. Asterisks indicate clinically meaningful changes (≥4 points) [27]. Triangles indicate patients who initiated DMT at the first evaluation.

CHOP-INTEND scores (range: 0–64; higher scores indicate better motor function) were assessed at both clinical evaluations, except for patient 2 (newborn at the intensive care unit) and patient 6 (initially evaluated outside the screening workflow). In the group with two *SMN2* copies, scores ranged from 10 (at 15 DoL) to 55 (at 26–28 DoL), whereas those with more than two copies showed higher scores (46 at 16 DoL to 62 at 30–37 DoL) (Table 1), consistent with milder phenotypes. Among the fourteen newborns with screen-positive results, three demonstrated a change greater than four points in CHOP-INTEND scores between the first and second clinical evaluations: two showed an increase (patients 4 and 5), whereas one exhibited a decrease (patient 1) (Figure 3). The score improvements observed in patients 4 and 5 are likely attributable to physiological neuromuscular development over time. A decline in motor scores was observed in three patients, all harboring two *SMN2* copies, presenting as asymptomatic at first and symptomatic at second evaluation (at the time of confirmatory result): patient 1, whose score decreased from 58 to 46 within two weeks; patient 7, whose score decreased from 55 to 52 over three weeks; and patient 11, whose score decreased from 53 to 51 in one week (Figure 3).

At the first clinical evaluation, 30.8% of patients (4/13) exhibited hypotonia and hypo- or areflexia, as well as movement loss. Bell-shaped chest, respiratory or bulbar impairment, paradoxical breathing, and tongue fasciculations were present at 23.08% (3/13) (Table 2). All cases that were symptomatic at the first evaluation (38.5%, 5/13) were males. By the second evaluation, the frequency of neuromuscular and respiratory findings increased: hypotonia rose to 50.0% (7/14), hypo- or areflexia to 57.1% (8/14), and both movement loss and tongue fasciculations to 35.7% (5/14). Respiratory or bulbar impairment was observed in 35.7% (5/14) of patients, and paradoxical breathing in 28.6% (4/14). In contrast, the bell-shaped chest remained relatively stable (23.1% to 21.4%). Notably, symptoms continued to be more prevalent among male infants overall. Only one patient (patient 14) presented with cardiac anomalies.

### 3.4. Genetic Counseling for Parents of Infants with 5q-SMA

Following molecular confirmation, genetic counseling was provided to 13 families; one family declined participation. A positive family history of 5q-SMA was reported in one case, while seven families noted relatives with other neurodevelopmental conditions, such as intellectual disability, autism spectrum disorder, or epilepsy. No consanguineous unions were reported. Regarding family structure, two infants were each members of dizygotic twin pairs from different families. Among the 13 families counseled, the parents in four cases and the father in an additional case chose not to undergo *SMN1/2* genotyping. Of the eight families that completed parental testing, both parents were carriers (*SMN1* heterozygous genotype) in five instances. Notably, in one family both parents carried two *SMN1* copies, and in two other families, the father was found to have two *SMN1* copies (Table A1). In such cases, a 2 + 0 genotype (silent carrier) could explain these findings, with frequencies varying substantially among populations (e.g., 3.63% in Caucasians and 27.51% in African Americans [28], for instance). Additionally, de novo variants, which account for approximately 1.0–3.4% of cases [29], and genetic mosaicism [30,31] were also addressed with the parents during the genetic counseling. It is important to note that the assessment and risk estimates were discussed in the context of the specific couple evaluated as biological parents, and this was clearly explained during genetic counseling.

## 4. Discussion

### 4.1. Timely Identification of 5q-SMA Through Newborn Screening

This is the first Brazilian 5q-SMA NBS pilot program to systematically report technical, clinical, and operational time indicators. The results show effective identification and early treatment (around one month of age) of infants with confirmed diagnosis, and integrated within the existing public health network. Moreover, the pilot data also provides evidence for the implementation of 5q-SMA in NBS programs in the context of São Paulo State, a reasonable genetic representation of the Brazilian population [32], and serves as a model for other regions. The results demonstrate the rapid progression of neuromuscular and respiratory impairment over a short follow-up period, which may be mitigated through systematic NBS, as newborns with screen-positive results show higher likelihoods of achieving independent standing and walking [9].

Importantly, given the severity and rapid progression of the disease, our findings suggest that this model may be particularly valuable when confirmatory testing is delayed. In such cases, referral for clinical evaluation should occur immediately after the screening real-time PCR, even before MLPA confirmation becomes available. This situation may occur when confirmatory testing is performed by an external reference laboratory with prolonged turnaround times, as observed in the present study. Under these circumstances, early evaluation by a specialized clinician may enable timely recognition of urgent clinical needs, such as respiratory muscle weakness in severe presentations, while also allowing the documentation required for access to DMT through the public healthcare system to be prepared before formal confirmatory results become available.

Regarding the first tier, samples from the city consistently showed shorter turnaround times, likely due to the proximity to the IJC Molecular Biology Laboratory. Nevertheless, indicators for both the city and the State of São Paulo demonstrated that all sample sources met the recommended screening timelines, and the timing of DMT initiation did not differ between patients from the capital and those from other municipalities. The prior implementation of the care pathway at the state level, supported by a multidisciplinary working group formally established by the São Paulo State Health Department [33], was fundamental to this achievement. This structure enabled coordinated action to ensure the timely referral of infants with screen-positive 5q-SMA results to the CERAME. Another Brazilian study has demonstrated the technical feasibility of incorporating 5q-SMA screening into the NBS workflow using the SALSA MC002 SMA Newborn Screen (MRC Holland) followed by MLPA confirmation [34]. However, aside from the challenges associated with performing automated analyses at a high-throughput scale [16], the aforementioned study was focused on prevalence determination and the time between birth and sample collection varied beyond the ideal 3 to 5 DoL, with at least one sample collected at 2 months of age, and first-tier results of confirmed patients at more than 1.5 months of age. On the other hand, the present study was based on real-life components with median time from birth to a screen-positive result of 11 DoL (9–16).

With 64.3% of infants asymptomatic at the time of the initial clinical evaluation, following a screen-positive result, the median time to confirmation in our pilot study (11 DoL) was comparable to other NBS programs worldwide. However, the timing of screen-positive results varies substantially across programs and countries. The Russian pilot reported the latest median time at 24 DoL (9–47) [35], while earlier identification was achieved in Poland with a median of 9 DoL (7–13) [36]. The fastest and most consistent timelines were observed in Australia and Canada, where first-tier results were available by approximately 8 DoL, with ranges of 5–18 and 5–13 DoL, respectively [37,38].

Regarding confirmatory tests, the median time from birth to confirmed 5q-SMA diagnosis also varied considerably across programs. In our study, confirmation was achieved at a median of 25 DoL (20–45), which was earlier than reported in Russia (median: 39 DoL; 19–69) [35], but later than in Canada and Germany, both achieving confirmation at a median of 14 DoL (12–24 and 9–23, respectively) [37,39]. Similarly early timelines were observed in Poland (median: 15 DoL; 11–19) and Australia (median: 18.5 DoL; 13–24) [36,40]. For the large-scale screening and treating of patients in Brazil, we found the socioeconomic and logistical factors to be the most relevant challenge on the way to the optimization of diagnosis confirmation. Delays from the MLPA supplier and/or difficulties in reaching out families with limitation of contact resulted in treatment initiation beyond a month of life in three cases (37, 42 and 49 DoL). To address this issue, one potential improvement would be the internalization of MLPA testing using DBS samples as a DNA source, as has already been successfully achieved by two others Brazilian NBS services (personal communication). With this approach, the turnaround time was reduced to approximately 48 h. Alternatively, patient support programs provided by pharmaceutical companies could facilitate partnerships with external laboratories (MLPA testing), helping to reduce turnaround times to fewer than six working days. For the first alternative, given the current sample volume, such an approach would not be cost-effective for a single NBS center; suggesting that extending coverage to the entire state (or even a country region) could make it more viable. Russia and Canada have also reported the challenge related to the second tier for treatment start regarding suboptimal logistics of samples and reagents [35,38]. Recent studies have proposed alternative approaches that may help optimize time to diagnosis. Blasco-Pérez et al. (2021) presented a rapid method for sequencing the entire *SMN2* gene, which enhances phenotype prediction and could support earlier therapeutic decisions when integrated into confirmatory testing [41]. In this study, the authors demonstrated that full-length *SMN2* sequencing allows the identification of intragenic variants, paralogous sequence variants, and *SMN1*–*SMN2* hybrid structures that are not detected by copy number-based assays. Importantly, the method can explain discordant genotype–phenotype correlations. By providing a more refined molecular characterization within a short turnaround time, this approach adds clinically relevant prognostic information beyond *SMN2* copy number alone and is particularly valuable in the context of NBS, where rapid and accurate risk stratification is essential for timely treatment initiation.

### 4.2. Population-Level SMN2 Copy Number Distribution and Symptom Severity

In our cohort, most individuals harbored two copies of *SMN2* (78.6%, 11/14). At the first clinical visit, 10 of these 11 individuals were evaluated, and half were symptomatic. By the second visit, all 11 had been evaluated, and the majority (81.8%, 9/11) presented symptoms. Among those with two *SMN2* copies, the first CHOP-INTEND score assessment showed a wide range, from 10 to 58. A previous study investigated if clinical findings at first examination following NBS were predictive of children’s outcome, and had indicated that CHOP-INTEND scores < 30 or >50 might indicate a less favorable prognosis or good prognosis, respectively, under early treatment, with score of 50 or higher indicating a high probability of acquiring independent sitting [42]. However, in the present study, our data challenge the applicability of early CHOP-INTEND thresholds as indicators of outcomes in patients carrying two copies of *SMN2*. Within this subgroup, short-term follow-up demonstrated that patients with CHOP-INTEND scores above 50 may exhibit numerical stability or mild worsening, including a marked decline of 10 points in one case (patient 1), indicating that high scores do not necessarily correspond to a favorable prognosis. Conversely, low CHOP-INTEND scores were not uniformly associated with unfavorable short-term trajectories, as numerical stability or improvement was observed in some cases. Moreover, integrating CHOP-INTEND with age at treatment initiation did not yield meaningful stratification, as early treatment occurred in both symptomatic and asymptomatic infants across all score ranges. Mild improvements, possibly reflecting typical motor development, were observed in an asymptomatic infant (patient 3). In addition to the limited sample size precluding formal statistical inference, longer-term follow-up is required to draw definitive conclusions. Taken together, these findings suggest that, in the context of NBS and very early assessment, CHOP-INTEND primarily reflects motor status at the time of assessment rather than clear prognosis after DMT. According to Cutrona et al. (2024), eight of the sixteen CHOP-INTEND items, including spontaneous limb movements, hand grasp, head control, and elbow flexion, are developmentally appropriate within the first weeks of life, whereas items assessing reaching or axial control become reliable only after the third month [43]. Therefore, apparent changes between evaluations may reflect both therapeutic effects and physiological neuromotor maturation. In this context, longitudinal interpretation of CHOP-INTEND scores in neonates should account for age assessment, developmental appropriateness of test items, and maturational baselines, enabling more accurate analyses in early-intervention settings.

Across NBS programs internationally, *SMN2* copy number distribution consistently clusters around two and three copies, with two copies being the most frequent category in several countries, including Germany [39], Belgium [44], Australia [40], Italy [45], Spain [46], and the São Paulo cohort in Brazil (this study). In contrast, three *SMN2* copies represent a substantial proportion of cases and are equally or more frequent than two copies in some programs, such as Russia [35], Canada [37], Serbia [47], and the combined Rio Grande do Sul and São Paulo sampling (Brazil) [34] (detailed comparison of international cohorts is provided in Table A2). This last finding might suggest that, compared to the present study, there is a potential regional genetic or demographic influence on *SMN2* copy number distribution in Brazil. Overall, one *SMN2* copy cases are uncommon, typically accounting for approximately 5–7% of cases in larger cohorts, as observed in Italy [45] and in the United States [48], although higher proportions may be seen in small cohorts, such as the Valencian Community in Spain [46]. Collectively, these findings support that most newborns with screen-positive results harbor two or three *SMN2* copies, corresponding to genotypes associated with early-onset forms of 5q-SMA, while the observed variability across countries likely reflects population-specific genetic and demographic factors.

### 4.3. Socioeconomic Factors Affecting Diagnostic Confirmation and Treatment Initiation

Among infants with confirmed 5q-SMA who were eligible to DMT (13/14), treatment was initiated at a median age of 28 DoL. Risdiplam was the most frequently administered therapy. In most cases, the initial doses were supplied through donations from non-profit parent organizations, reflecting the convenient oral administration and rapid availability of Risdiplam. Across NBS programs, the timing of treatment initiation varies substantially worldwide. Early-start programs typically initiate DMT within the first 2–4 weeks of life, enabling presymptomatic treatment for nearly all infants with confirmed diagnosis, as demonstrated in Canada [37,38] and Australia [40], although 40% of patients exhibited symptoms within the first week of life in the last-mentioned country. In contrast, later initiation, occurring at a median of approximately 3 months of age, is associated with a higher proportion of symptomatic infants at first clinical evaluation, as reported in Russia [35]. Additionally, logistical barriers, such as prolonged sample transportation, can significantly delay treatment initiation, as observed in Osaka, Japan [49]. Socioeconomic factors may also contribute to delays in initiating non-invasive ventilation, which can lead to hospitalization for infections or respiratory failure in symptomatic patients, potentially resulting in worsening motor function, reflected in CHOP-INTEND score. These findings underscore the importance of coordinated public health policies, including the area of rehabilitation. Collectively, these findings reinforce that earlier treatment initiation, ideally within the first month of life, represents the optimal therapeutic window, maximizing presymptomatic intervention, and improving motor outcomes.

Notably, although there is a global consensus among specialists supporting treatment for all infants with confirmed 5q-SMA carrying four or fewer copies of *SMN2*, [50], current Brazilian public health policies diverge from this recommendation by restricting treatment eligibility to patients with up to three *SMN2* copies [51]. Consequently, as previously mentioned, one patient in the present study was not eligible for DMT due to having four *SMN2* copies. This discrepancy forces a “wait and see” approach that often triggers significant psycho-emotional distress for families, as the anxiety of facing a confirmed diagnosis is compounded by the lack of immediate therapeutic options [52].

From the public health resource management perspective, a recent Brazilian study demonstrated through cost-utility analysis that screening for 5q-SMA is a cost-effective and a dominant strategy when compared with the absence of screening. The model estimates a positive annual impact of approximately BRL 68 million in savings for the healthcare system, mainly driven by the reduction in lifetime costs related to medications and medical care, considering an annual cohort of approximately 2.5 million live births in Brazil [53]. Beyond its economic implications, the strategy was associated with substantial clinical gains. In the modeled population, NBS resulted in 691 additional life-years (LYs) and 965 additional quality-adjusted life-years (QALYs), increasing total QALYs from 2367 without screening to 3332 with screening implementation. These findings reflect a meaningful extension of survival accompanied by improved health-related quality of life. When expressed in more tangible terms, the projected benefit corresponds to more than 250 thousands of additional DoL and approximately 350 thousands of days lived with better quality compared with a non-screening strategy. These gains are concentrated in the approximately 250 children with 5q-SMA born each year in Brazil, reflecting the cumulative impact of early diagnosis and treatment rather than an isolated benefit per individual. Previously published data in the scientific literature corroborate the Brazilian model and also demonstrate that NBS is a sustainable intervention, with the potential to significantly reduce costs associated with prolonged hospitalizations, assisted ventilation, intensive rehabilitation, and loss of family productivity, while offering affected children the opportunity to achieve important motor milestones and a life with greater autonomy and economic productivity through effective social participation. In a comparable analysis, an Australian study showed that the screening strategy resulted in a lifetime gain of 267 QALYs and incremental savings of USD 3.98 million, corresponding on average to 9 additional QALYs and savings of USD 134.6 thousand per 5q–SMA case [54]. In addition, the model indicates that each dollar invested in NBS yields savings of USD 3.69 for the hospital system. In Italy, the implementation of screening generated an increase of 390 QALYs per annual birth cohort, in addition to lifetime savings of € 1,513,375 [55]. In the United Kingdom, the benefits were even greater, with 529 additional QALYs and an estimated cost reduction of £ 62,191,531.00 [56].

Taken together with present pilot evidence, these data reinforce that early diagnosis and timely access to DMT produce relevant clinical benefits and more efficient use of healthcare resources.

## 5. Conclusions

The 5q-SMA NBS pilot program in São Paulo, Brazil, demonstrated that coordinated action among public health authorities, specialized referral centers, and diagnostic laboratories within a defined jurisdiction enables timely diagnostic confirmation and early initiation of intervention in newborns with screen-positive results. The estimated birth prevalence was consistent with international reports, and the distribution of *SMN2* copy number revealed a predominance of newborns with two copies, supporting the clinical relevance of early detection in preventing early morbidity and mortality. The program achieved high clinical specificity and was successfully integrated into routine dried blood spot-based NBS workflows.

Although the time from screening to intervention was clinically meaningful, opportunities for optimization remain. Delays related to confirmatory testing logistics and socioeconomic barriers affecting follow-up attendance were identified as key implementation challenges. The operational evidence generated, including indicators for specimen collection, transport, result reporting, and short-term follow-up, supports the feasibility of expanding 5q-SMA NBS nationwide and strengthens the foundation for implementation of Law 14,154/2021 within the Brazilian NBS system.

## Figures and Tables

**Table 1:** Clinical and genetic characterization of infants with screen-positive results. Motor function was assessed via CHOP-INTEND (range: 0–64); genotypes were determined by MLPA. Age values are reported in days of life (DoL). Clinical status was assessed by specialists at the CERAME.

Patient	*SMN2* Exon 7/Exon 8		1st Evaluation			2nd Evaluation			Age at DMT Initiation
			Status	Score	Age	Status	Score	Age	
P1	2	2	Asymptomatic	58	13	Symptomatic	46	27	28
P2	2	2	—	—	—	Asymptomatic	55	26	27
P3	2	1	Asymptomatic	46	16	Asymptomatic	48	23	23
P4	2	1	Symptomatic	29	17	Symptomatic	39	24	17
P5	3	3	Asymptomatic	46	17	Asymptomatic	62	31	31
P6 *	2	2	Symptomatic	-	3	Symptomatic	24	31	5
P7	2	2	Asymptomatic	55	28	Symptomatic	52	49	49
P8	2	1	Symptomatic	32	15	Symptomatic	32	22	24
P9	2	1	Symptomatic	52	42	Symptomatic	52	63	42
P10	3	3	Asymptomatic	62	30	Asymptomatic	62	37	37
P11	2	2	Asymptomatic	53	21	Symptomatic	51	28	28
P12	2	2	Asymptomatic	10	15	Symptomatic	10	29	29
P13	4	4	Asymptomatic	60	15	Asymptomatic	61	29	—
P14	2	2	Symptomatic	30	19	Symptomatic	31	33	19

* First evaluated prior to the NBS program; —, data not available.

**Table 2:** Distribution of symptoms in the cohort of newborns with screen-positive results. First and second evaluations occurred after first-tier and confirmatory results, respectively. Data were obtained by neuromuscular specialists at the CERAME.

Symptom	First Clinical Evaluation (*n* = 13)	Second Clinical Evaluation (*n* = 14)
Hypotonia	30.8% (*n*= 4 males)	50% (*n* = 5 males, 2 females)
Hypo or areflexia	30.8% (*n* = 4 males)	57.1% (*n* = 6 male, 2 females)
Movement loss	30.8% (*n* = 4 males)	35.7% (*n* = 4 males, 1 female)
Tongue fasciculation	23.1% (*n* = 3 males)	35.7% (*n* = 4 males, 1 female)
Bell-shaped chest	23.1% (*n* = 3 males)	21.4% (*n* = 3 males)
Respiratory/bulbar impairment	23.1% (*n* = 3 males)	35.7% (*n* = 4 males, 1 female)
Paradoxical breathing	23.1% (*n* = 3 males)	28.6% (*n* = 3 males, 1 female)

## Data Availability

The datasets generated and analyzed during the current study are available from the corresponding author on reasonable request due to privacy and ethical restrictions involving genetic and familial data.

## Abbreviations

The following abbreviations are used in this manuscript:

5q-SMA	5q spinal muscular atrophy
CAAE	Certificado de Apresentação de Apreciação Ética (Certificate of Presentation for Ethical Approval)
CERAME	Centros Especializados de Referências de Atrofia Muscular Espinhal (Specialized Reference Centers for Spinal Muscular Atrophy)
CHOP-INTEND	Children’s Hospital of Philadelphia Infant Test of Neuromuscular Disorders
CONEP	Comissão Nacional de Ética em Pesquisa (National Commission for Research Ethics)
DBS	dried blood spots
DMT	disease-modifying therapy
DoL	days of life
dPCR	digital polymerase chain reaction
IJC	Instituto Jô Clemente (Jô Clemente Institute)
IQR	interquartile range
KREC	kappa-deleting recombination excision circles
LGPD	Lei Geral de Proteção de Dados (Brazilian General Data Protection Law)
MLPA	multiplex ligation-dependent probe amplification
NBS	newborn screening
OMIM	Online Mendelian Inheritance in Man
real-time PCR	real-time polymerase chain reaction
RSNS	Reference Services for Neonatal Screening
SMN	survival motor neuron
*SMN1*	survival motor neuron 1
*SMN2*	survival motor neuron 2
TREC	T-cell Receptor Excision Circles

## Appendix B

**Table A1:** Genetic counseling outcomes and parental genotypes. Family history and sibling categories were obtained during counseling. *SMN1* and *SMN2* exon 7 copy numbers were determined by MLPA in parental samples. Data are presented for the 11 families who participated in genetic counseling out of the 14 cases identified in the pilot program; all information is presented in an anonymized form.

Patient	*SMN1*			*SMN2*		Family History	Siblings
	M	P		M	P		
P1	1	1		3	1	None	≥1 half-sibling
P2	1	1		2	1	Neurological disorder in extended family	Multiple siblings
P4	1	1		2	2	Neurodevelopmental disorders in extended family	None
P5	1	2		1	3	Neurodevelopmental disorders in extended family	≥1 sibling
P6	—	—		—	—	Positive family history of 5q-SMA	≥1 sibling
P7	1	1		1	2	Delayed motor development in family	Multiple siblings
P9	2	2		1	1	Neurodevelopmental disorders in extended family	≥1 half-sibling
P10	1	—		2	—	Neurodevelopmental disorders in extended family	≥1 half-sibling
P11	1	1		2	2	Delayed motor development in family	None
P13	1	2		2	3	None	≥1 sibling

M, maternal genotype; P, paternal genotype.

## Appendix C

**Table A2:** Distribution of *SMN2* copy number among confirmed 5q-SMA cases identified through national newborn screening programs by country. The table summarizes the number of newborns screened, confirmed cases, distribution (count and percentage) of *SMN2* copy number categories, estimated disease incidence, and corresponding references.

Country/Region	Newborns Screened	Confirmed Cases	*SMN2* Copy Number					Incidence	Reference
			1	2	3	4	≥5		
Brazil (São Paulo)	194,715	14	—	11 (78.6%)	2 (14.3%)	1 (7.1%)	—	1:13,908	This study
Brazil (Rio Grande do Sul & São Paulo)	40,000	4	—	1 (25.0%)	2 (50.0%)	1 (25.0%)	—	1:10,000	[34]
Canada (Ontario)	139,800	5	—	1 (20.0%)	3 (60.0%)	1 (20.0%)	—	1:27,960	[37]
United States (48 states)	6,244,825	425 *	12 (5.0%)	118 (49.2%)	79 (32.9%)	31 (12.9%)	—	1:14,694	[48]
Australia (New South Wales & Australian Capital Territory)	103,903	9	—	6 (66.7%)	3 (33.3%)	—	—	1:11,544	[40]
Russia (8 federal subjects)	202,908	26	—	10 (38.5%)	11 (42.3%)	4 (15.4%)	1 (3.8%)	1:7804	[35]
Taiwan (Zhongzheng)	120,267	7	—	3 (42.9%)	2 (28.6%)	2 (28.6%)	—	1:17,181	[57]
Belgium (Wallonia & Brussels)	136,339	10	—	5 (50.0%)	3 (30.0%)	2 (20.0%)	—	1:13,634	[44]
Germany (Bavaria & North Rhine-Westphalia)	297,163	43	—	17 (39.5%)	10 (23.3%)	14 (32.6%)	2 (4.7%)	1:6910	[39]
Italy (Lazio & Tuscany)	90,885	15	1 (6.7%)	8 (53.3%)	4 (26.7%)	1 (6.7%)	1 (6.7%)	1:6059	[45]
Spain (Valencian Community)	31,560	4	1 (25.0%)	3 (75.0%)	—	—	—	1:7890	[46]
Serbia (Belgrade)	54,393	6	—	2 (33.3%)	3 (50.0%)	1 (16.7%)	—	1:9066	[47]

* *SMN2* copy number data were available for 240 out of 425 confirmed cases [48]; the denominator of 240 was used for percentage calculations.
